# Supplementary material for: A fault-intrusion-tolerant system and deadline-aware algorithm for scheduling scientific workflow in the cloud
Source: PeerJ Comput Sci. 2021 Nov 2;7:e747. doi: 10.7717/peerj-cs.747 (PMC8576554; doi:10.7717/peerj-cs.747)
Supplement: Supplemental Information 6 [file peerj-cs-07-747-s006.zip › Myproject1.1/docs/org/cloudbus/cloudsim/VmAllocationPolicy.html]

VmAllocationPolicy


JavaScript is disabled on your browser.


- Overview
- Package
- Class
- Use
- Tree
- Deprecated
- Index
- Help

- Prev Class
- Next Class

- Frames
- No Frames

- All Classes

- Summary:
- Nested |
- Field |
- Constr |
- Method

- Detail:
- Field |
- Constr |
- Method


org.cloudbus.cloudsim

## Class VmAllocationPolicy

- java.lang.Object
- - org.cloudbus.cloudsim.VmAllocationPolicy

- Direct Known Subclasses:
  :   NetworkVmAllocationPolicy, PowerVmAllocationPolicyAbstract, VmAllocationPolicySimple

  ---

    

  ```
  public abstract class VmAllocationPolicy
  extends java.lang.Object
  ```

  VmAllocationPolicy is an abstract class that represents the provisioning policy of hosts to
  virtual machines in a Datacentre. It supports two-stage commit of reservation of hosts: first, we
  reserve the host and, once commited by the user, it is effectivelly allocated to he/she

  Since:
  :   CloudSim Toolkit 1.0

- - ### Constructor Summary

    Constructors

    Constructor and Description

    `VmAllocationPolicy(java.util.List<? extends Host> list)`

    Allocates a new VmAllocationPolicy object.
  - ### Method Summary

    Methods

    | Modifier and Type | Method and Description |
    | --- | --- |
    | `abstract boolean` | `allocateHostForVm(Vm vm)` Allocates a host for a given VM. |
    | `abstract boolean` | `allocateHostForVm(Vm vm, Host host)` Allocates a specified host for a given VM. |
    | `abstract void` | `deallocateHostForVm(Vm vm)` Releases the host used by a VM. |
    | `abstract Host` | `getHost(int vmId, int userId)` Get the host that is executing the given VM belonging to the given user. |
    | `abstract Host` | `getHost(Vm vm)` Get the host that is executing the given VM belonging to the given user. |
    | `<T extends Host>  java.util.List<T>` | `getHostList()` Gets the host list. |
    | `abstract java.util.List<java.util.Map<java.lang.String,java.lang.Object>>` | `optimizeAllocation(java.util.List<? extends Vm> vmList)` Optimize allocation of the VMs according to current utilization. |
    | `protected void` | `setHostList(java.util.List<? extends Host> hostList)` Sets the host list. |

    - ### Methods inherited from class java.lang.Object

      `clone, equals, finalize, getClass, hashCode, notify, notifyAll, toString, wait, wait, wait`

- - ### Constructor Detail


    - #### VmAllocationPolicy

      ```
      public VmAllocationPolicy(java.util.List<? extends Host> list)
      ```

      Allocates a new VmAllocationPolicy object.

      Parameters:
      :   `list` - Machines available in this Datacentre
  - ### Method Detail


    - #### allocateHostForVm

      ```
      public abstract boolean allocateHostForVm(Vm vm)
      ```

      Allocates a host for a given VM. The host to be allocated is the one that was already
      reserved.

      Parameters:
      :   `vm` - virtual machine which the host is reserved to

      Returns:
      :   $true if the host could be allocated; $false otherwise


    - #### allocateHostForVm

      ```
      public abstract boolean allocateHostForVm(Vm vm,
                              Host host)
      ```

      Allocates a specified host for a given VM.

      Parameters:
      :   `vm` - virtual machine which the host is reserved to

      Returns:
      :   $true if the host could be allocated; $false otherwise


    - #### optimizeAllocation

      ```
      public abstract java.util.List<java.util.Map<java.lang.String,java.lang.Object>> optimizeAllocation(java.util.List<? extends Vm> vmList)
      ```

      Optimize allocation of the VMs according to current utilization.

      Parameters:
      :   `vmList` - the vm list
      :   `utilizationBound` - the utilization bound
      :   `time` - the time

      Returns:
      :   the array list< hash map< string, object>>


    - #### deallocateHostForVm

      ```
      public abstract void deallocateHostForVm(Vm vm)
      ```

      Releases the host used by a VM.

      Parameters:
      :   `vm` - the vm


    - #### getHost

      ```
      public abstract Host getHost(Vm vm)
      ```

      Get the host that is executing the given VM belonging to the given user.

      Parameters:
      :   `vm` - the vm

      Returns:
      :   the Host with the given vmID and userID; $null if not found


    - #### getHost

      ```
      public abstract Host getHost(int vmId,
                 int userId)
      ```

      Get the host that is executing the given VM belonging to the given user.

      Parameters:
      :   `vmId` - the vm id
      :   `userId` - the user id

      Returns:
      :   the Host with the given vmID and userID; $null if not found


    - #### setHostList

      ```
      protected void setHostList(java.util.List<? extends Host> hostList)
      ```

      Sets the host list.

      Parameters:
      :   `hostList` - the new host list


    - #### getHostList

      ```
      public <T extends Host> java.util.List<T> getHostList()
      ```

      Gets the host list.

      Returns:
      :   the host list


- Overview
- Package
- Class
- Use
- Tree
- Deprecated
- Index
- Help

- Prev Class
- Next Class

- Frames
- No Frames

- All Classes

- Summary:
- Nested |
- Field |
- Constr |
- Method

- Detail:
- Field |
- Constr |
- Method
